# Application of the Ecohealth Model to Translate Knowledge into Action in the Health Sciences

**DOI:** 10.1289/ehp.1104847

**Published:** 2012-03-01

**Authors:** Armando Arredondo, Emanuel Orozco

**Affiliations:** Center for Health Systems Research, National Institute of Public Health, Cuernavaca, Morelos, Mexico, E-mail: armando.arredondo@insp.mx

As noted by Barkin and Schlundt (2011), addressing the public health needs of the population using evidence from biomedical research necessarily requires a wholistic approach that is both multilevel and multidisciplinary. Although there may be public health benefits, there are also important challenges when generating knowledge, at the microenvironmental level, as well as at the macroenvironmental level. This happens particularly when evidence is translated into interventions that generate benefits for all who are involved in the health process; for example, in dealing with obesity, these interventions would benefit users, the health system, food producers, and others. To complement the response to these challenges, we suggest a greater application of the ecohealth model. This model has been proposed as a new analytical model for research action based on the ecosystemic approach to human health, an approach that places health within the realm of the environment and acknowledges cause–effect interconnections between human health and humans’ biophysical, social, and economic environment.

The ecohealth model stems from the generation of health knowledge and the multiple interconnections between the different components of the ecosystem. It sets forth that these interconnections are complex and interdependent and include social determinants and disparities, as well as biophysical determinants. From this perspective, scientists need to revise their models and research methods and open up to new analytical focuses and new forms of collaboration and interaction, going beyond the biophysical characteristics of systems and the scientific community itself. For many reasons, the traditional methods used in the study of the micro–macro environment have not been able to fulfill the expectations for health and welfare or those for improving sanitary conditions of populations. Thus, we need to periodically evaluate evaluations and adjust programs, interventions, and health policies.

Although traditional methods take into account the economy and the community, often at the expense of the environment (jeopardizing the possibility of a sustainable ecosystem), the ecohealth model breaks up each of its components into different categories (Hancock 1990; Lebel 2005). It confers equal importance to environmental management, economic factors, and the community’s aspirations, and it places human health at the center of the intersection of these three elements. In this sense, the ecohealth model itself is part of the sustainable development process, and its fundamental premise is to be inclusive. Interventions and health programs based on evidence generated under the ecohealth model should be more cost-effective than many medical treatments or traditional healthcare interventions. This analytical model and its methodological research approach involve three participating groups: researchers and other specialists; community members, such as common citizens, businessmen, farmers, fishermen, and miners; and decision makers in health interventions. Besides the need for the participation of these three groups, the ecohealth model is based on three methodological pillars: transdisciplinarity, participation and equity.

- Transdisciplinarity implies a multilevel and translevel vision, with a broad scope and collaboration in the study of health determinants and conditions related to the ecosystem.
- Participation intends to achieve consensus on the definition of the study’s objective among scientists, community members, and decision makers, both between and within groups.
- Equity includes the analysis of the roles of men and women and their different degrees of influence in decisions on access to and use of financial resources, as well as equity in benefits and rewards for all of those involved in a concrete health problem.

Each of these pillars generates, to a great extent, conditions for a more effective and efficient translation of scientific knowledge into action, as well as addressing the challenges set forth by Barkin and Schlundt (2011). In fact, the transdisciplinarity component responds to the need for greater collaboration between researchers from all involved disciplines, as well as other social actors studying the same problem. Participation and equity in the involved groups (researchers, decision makers, and community members) at the time of implementing interventions and generating benefits would guarantee a greater effectiveness in the design and implemention of interventions with an ecohealth focus. These components would also promote greater equality and equity in the benefits and rewards for all involved parties.

In summary, the collaboration between researchers during generation and application of scientific evidence—at both the microenvironmental and macroenvironmental levels—can guarantee greater benefits, acceptability, and effectiveness in interventions at the population level for all of those involved.
